# Oxidative Stress and Cancer: Advances and Challenges

**DOI:** 10.1155/2016/5010423

**Published:** 2016-05-16

**Authors:** Sahdeo Prasad, Subash C. Gupta, Manoj K. Pandey, Amit K. Tyagi, Lokesh Deb

**Affiliations:** ^1^University of Texas MD Anderson Cancer Center, 1901 East Road, Houston, TX 77054, USA; ^2^Department of Biochemistry, Institute of Science, Banaras Hindu University, Varanasi, Uttar Pradesh 221 005, India; ^3^Penn State Milton S. Hershey Medical Center, 1214 Research Boulevard, Hummelstown, PA 17036, USA; ^4^Institute of Bioresources and Sustainable Development (IBSD), Takyelpat Institutional Area, Imphal, Manipur 795 001, India

Reactive oxygen species (ROS) constantly generated inside the body are required to drive regulatory pathways and are also a cause for several pathological conditions including cancer. Numerous lines of evidence suggest that ROS can promote as well as suppress the survival of cancer cells.* First*, ROS are known to regulate each and every step of tumor development including transformation, survival, proliferation, invasion, metastasis, and angiogenesis.* Second*, chronic inflammation, one of the major mediators of cancer, is regulated by ROS.* Third*, ROS are known to regulate signaling molecules required for cell cycle progression.* Fourth*, the expression of various tumor suppressor genes is under control of ROS.* Fifth*, a high level of ROS can suppress tumor growth through the sustained activation of the cell cycle inhibitors.* Sixth*, most of the currently available chemotherapeutic and radiotherapeutic agents kill cancer cells by increasing ROS stress. Thus, both ROS-elevating and ROS-eliminating strategies have been developed for cancer therapy.

This special issue is an effort to assess the existing concepts, recent findings, controversies, and challenges concerning the role of ROS in tumor development. In particular, the topics covered in this special issue include understanding the role of ROS in cancer initiation and progression (M. Tafani et al.), cancer cell signaling (M. Tafani et al., H. S. Khalil et al., I. Ryoo et al., L. Zong et al., and J. H. Osaki et al.), drug resistance (A. Barreiro-Alonso et al.), autophagy (L. Zhang et al.), and cancer therapy (N. Mut-Salud et al., A. M. Mileo and S. Miccadei, A. Jarosz et al., and Z.-G. Jiang et al.). The article by A. Lyakhovich and M. E. Lleonart discusses various mechanisms developed by cancer stem cells to attenuate ROS levels. Another article deals with the role of ROS in inducing polycystic ovary syndrome (PCOS), a disease condition in women associated with an increasing risk of cancers (T. Zuo et al.). The role of ROS in numerous cancer types including breast (A. C. S. A. Herrera et al., A. L. G. Júnior et al.), ovarian (A. Barreiro-Alonso et al.), prostate (A. Barreiro-Alonso et al.), hepatocellular carcinoma (B. K. Maurya and S. K. Trigun, Y.-Q. Hou et al.), pancreatic cancer (L. Zhang et al. and L. Zong et al.), bladder cancer (N. V. Savina et al.), colorectal cancer (J. Kabzinski et al.), and cervical cancer (J. H. Osaki et al.) is discussed.

It is our hope that these articles will be useful to the readers.

